# A neuropsychiatric presentation of suspected immune-mediatedneuropathy: diagnostic overlap between Guillain–Barré syndrome andautoimmune encephalitis—a case

**DOI:** 10.1186/s13256-026-06423-y

**Published:** 2026-07-30

**Authors:** Christopher Washington, Alexander Morris, Natalia Ortiz

**Affiliations:** 1 Lewis Katz School of Medicine, Temple University, Philadelphia, USA; 2 Department of Neurology, Temple University Hospital, Philadelphia, United States; 3 Present Address: Division of Neurocritical Care, Department of Neurology, Penn Medicine, University of Pennsylvania Health System, Philadelphia, United States; 4 Department of Psychiatry and Behavioral Science, Lewis Katz School of Medicine at Temple University; Temple University Hospital, Philadelphia, United States

**Keywords:** Guillain–Barré syndrome, Neuropsychiatry, Psychopathology, Autoimmune encephalopathy, Case report

## Abstract

**Background:**

Guillain–Barré syndrome (GBS) is an acute, immune-mediated polyneuropathy classically characterized by rapidly progressive, symmetrical limb weakness and areflexia. Although neuropsychiatric manifestations have been described, they remain uncommon and may lead to diagnostic delay or misattribution to primary psychiatric illness.

**Case presentation:**

We describe a 58-year-old woman with a history of rheumatoid arthritis who presented with hematemesis secondary to gastritis and altered mental status (AMS), preceded by several weeks of progressive behavioral changes, including withdrawal, dysphoria, hallucinations, and disorientation. During hospitalization, her mental status continued to decline despite trials of antipsychotic and benzodiazepine therapy. Neurological examination revealed progressive ascending weakness, areflexia, and sensory deficits. Cerebrospinal fluid analysis demonstrated albuminocytologic dissociation. In the absence of electrophysiological confirmation, the findings raised concern for an immune-mediated polyradiculoneuropathy such as Guillain–Barré syndrome; however, definitive classification was not possible because electrophysiological testing was not performed. The patient was treated with a five-day course of intravenous immunoglobulin (IVIG), after which both motor function and mental status improved; however, treatment response was interpreted cautiously because IVIG is not disease-specific.

**Conclusion:**

This case highlights the diagnostic complexity of neuropsychiatric presentations in suspected GBS. The prominence and early onset of psychiatric symptoms necessitated a broad differential diagnosis, including delirium, metabolic encephalopathy, autoimmune encephalitis, and acute porphyria. Additionally, the patient’s history of rheumatoid arthritis raised consideration of alternative immune-mediated neuropathies, including vasculitic neuropathy. Importantly, autoimmune encephalitis could not be excluded due to the absence of neuroimaging, EEG, and antibody testing. Furthermore, clinical improvement following IVIG is not disease-specific and may occur in multiple autoimmune conditions. This case underscores the diagnostic tension between peripheral and central nervous system autoimmune processes in the setting of incomplete diagnostic data. GBS should remain a consideration in patients presenting with concurrent neurological and psychiatric symptoms; however, caution is warranted when diagnostic studies are limited. This case emphasizes the importance of maintaining a broad differential diagnosis and highlights the potential for overlap between peripheral neuropathies and central autoimmune encephalopathies. Early multidisciplinary evaluation and careful interpretation of treatment response are essential for optimizing patient outcomes. This report should be interpreted as an educational case illustrating the diagnostic complexity of overlapping neuropsychiatric and peripheral neurological manifestations rather than as definitive evidence of an atypical presentation of Guillain–Barré syndrome.

**Supplementary Information:**

The online version contains supplementary material available at https://doi.org/10.1186/s13256-026-06423-y.

## Background

Guillain–Barré syndrome (GBS) is an autoimmune disorder characterized by rapidly progressive, symmetrical weakness of the extremities. GBS has an incidence that ranges from 0.81 to 1.91 cases per 100,000 person-years (median 1.11) in North America, with a 20% increase in incidence observed with every 10-year increase in age. Of the various documented subtypes, the most common is acute inflammatory demyelinating polyneuropathy (AIDP). Despite its relatively low incidence, GBS remains an essential component of the differential diagnosis for patients presenting with acute neurological deficits [1].

It has been previously shown that atypical manifestations may delay the diagnosis of GBS. Unfortunately, it can take an affected patient up to five Emergency Department visits before GBS is even considered [2]. GBS, despite being a primarily motor neuropathy, can present with related sensory disturbances that are also a component of its formal diagnosis [3]. These disturbances may range from pain to the more commonly occurring paresthesias. AIDP rarely demonstrates alterations in cognition, nonspecific altered mental status (AMS), and/or hallucinations; nonetheless, several reports indicate that such associations may occur [4].

Thorough neurologic evaluation is associated with improvement in both time of diagnosis and patient condition on discharge. Patients with delays in diagnosis and treatment are substantially more likely to face severe complications, including respiratory paralysis [2, 4 ]. Approximately 30% of GBS patients become reliant on mechanical ventilation; accordingly, about 75% of these patients subsequently develop ventilator-associated pneumonia. Other serious complications include sepsis, pulmonary embolism, and cardiac arrest [5]. Furthermore, approximately 20% of GBS patients will sustain some degree of long-term disability [6–8].

Recognizing GBS as a predominantly clinical diagnosis, coupled with its diverse and potentially life-threatening complications, underscores the importance of carefully documenting atypical presentations. We present a diagnostically complex case of suspected immune-mediated neuropathy in which prominent neuropsychiatric symptoms evolved alongside objective peripheral nervous system findings concerning for an immune-mediated polyradiculoneuropathy, including Guillain–Barré syndrome, while autoimmune encephalitis and other competing diagnoses could not be definitively excluded.

## Case presentation

A 58-year-old female with a past medical history significant for an unverified schizophrenia diagnosis and rheumatoid arthritis was admitted following the onset of nausea, hematemesis, subjective fevers, and altered mental status (AMS). A formal patient timeline is described narratively due to the retrospective nature of the case. Because the report is retrospective and initial neurological complaints were incompletely documented, the timeline below summarizes the temporal evolution of symptoms, neurological findings, diagnostic evaluation, and treatment (Table 1). Available records did not clearly document gait instability, falls, or limb weakness at the time of initial presentation. The earliest reported symptoms were gastrointestinal complaints, subjective fevers, altered mental status, and several weeks of behavioral change. Objective peripheral nervous system findings, including progressive weakness, sensory deficits, and areflexia, became evident later during hospitalization, prompting neurological consultation.

**Table 1:** Chronological summary of symptom evolution, neurological findings, diagnostic evaluation, and treatment course

Time point	Neuropsychiatric/systemic features	Neurological findings	Diagnostic/treatment events
~ 6 weeks before admission	Social withdrawal, dysphoria, irritability, abnormal sleep, cognitive decline, anhedonia	No documented limb weakness, gait instability, or falls in the reviewed medical record	Family reported the patient was previously independent with all activities of daily living
Admission	Nausea, hematemesis, subjective fevers, altered mental status	No documented initial peripheral motor complaints	Esophagogastroduodenoscopy (EGD) demonstrated gastritis
Early hospitalization	Hallucinations, internal preoccupation, social withdrawal; urinary tract infection treated	Peripheral neurological deficits not yet clearly documented	Trial of antipsychotic therapy without clinical improvement
Catatonia evaluation	Mutism, stupor, staring, withdrawal; lorazepam trial ineffective	–	Catatonia considered less likely
Neurology consultation	Persistent disorientation	Decreased muscle tone, lower-extremity sensory loss, diminished upper-extremity reflexes, absent lower-extremity reflexes	Lumbar puncture demonstrated elevated cerebrospinal fluid protein with normal white blood cell count
Two days later	Altered mental status persisted	Minimal finger movement with diffuse areflexia	Suspected immune-mediated polyradiculoneuropathy considered
IVIG day 2–3	Hallucinations resolved; orientation improved	Improved movement and muscle tone with partial return of reflexes and sensation	Continued intravenous immunoglobulin (IVIG) therapy
Discharge / follow-up	Returned to baseline mental status according to family	Residual weakness persisted	Discharged to skilled nursing facility; subsequently died from sepsis secondary to a decubitus ulcer

The patient’s history of rheumatoid arthritis introduces additional diagnostic complexity. However, details regarding disease severity and immunosuppressive therapy were unavailable, limiting assessment of infection risk, autoimmune overlap, and treatment-related complications.

The patient’s episode of hematemesis was evaluated with esophagogastroduodenoscopy (EGD), which demonstrated gastritis. While this provides a structural explanation for the bleeding, the extent of blood loss and its potential contribution to altered mental status (for example, via anemia or hypoperfusion) could not be fully determined due to limited laboratory and hemodynamic data.

The retrospective record available to the authors did not include retrievable values for Complete Blood Count (CBC), comprehensive metabolic panel, inflammatory markers, rheumatologic serologies, infectious studies, admission vital signs, or detailed pre-admission medication history, including immunosuppressive therapy and other medications potentially associated with neuropathy. Clinical documentation described routine laboratory evaluation as unremarkable relative to the presentation, but precise values could not be independently verified.

Regarding the schizophrenia diagnosis, it was not endorsed by family members, and there was ambiguity regarding how and when the diagnosis was made, as well as its overall accuracy. According to the patient’s daughter, approximately six weeks prior to admission, the patient was independently performing all activities of daily living. The daughter expressed concern that the patient had appeared progressively depressed prior to admission, exhibiting social withdrawal, irritability, abnormal sleep patterns, persistent dysphoria, cognitive decline, and anhedonia.

Early in hospitalization, the patient spoke only Spanish, experienced hallucinations, appeared preoccupied with internal stimuli, and gradually became socially withdrawn. Accounting for the possibility of a confounding presentation of schizophrenia and/or hypoactive delirium, aripiprazole was administered for several days. It was later discontinued due to lack of efficacy and undesirable side effects. A comprehensive delirium workup revealed a urinary tract infection (UTI), which was treated; however, the patient’s mental deterioration persisted.

Evaluation for catatonia demonstrated a Bush–Francis score (BFS) of 9, with points attributed to immobility/stupor, mutism, staring, and withdrawal. A lorazepam treatment trial did not result in improvement, reducing the likelihood of catatonia. Given the persistence of multisensory hallucinations and declining mentation, a primary psychiatric etiology was considered less likely, and an indeterminate delirium was proposed. Fluoxetine was continued due to mild improvement in mood.

Neuroimaging was significantly limited due to persistent patient non-cooperation despite pharmacologic attempts with lorazepam and antipsychotic agents. As a result, brain MRI and EEG could not be obtained. The reviewed medical record did not document whether head Computed Tomography (CT) was performed; if not, this represents an additional limitation, as structural, vascular, or inflammatory intracranial processes could not be formally excluded. The reviewed medical record also did not document whether spinal Magnetic Resonance Imaging (MRI) was performed; therefore, structural spinal pathology could not be fully evaluated.

Several diagnostic studies that would have strengthened differentiation between peripheral and central autoimmune disease were not completed. Brain MRI was not obtained because of persistent non-cooperation despite pharmacologic attempts. EEG was likewise not obtained. Serum and Cerebrospinal Fluid (CSF) autoimmune encephalitis antibody testing, including anti-NMDAR, LGI1, CASPR2, GABABR, and AMPAR antibodies, was not available in the reviewed record. Electrophysiological testing with Nerve Conduction Studies/Electromyography (NCS/EMG) was also not completed. These omissions substantially limited the ability to distinguish GBS from autoimmune encephalitis, paraneoplastic syndromes, vasculitic neuropathy, critical illness neuropathy, or other mimics.

Lumbar puncture revealed elevated CSF protein (126 mg/dL) with normal glucose (45 mg/dL) and White Blood Cell (WBC) count (1/mm3). Neurological examination demonstrated disorientation to place and event, spontaneous upper-extremity movement, decreased upper-extremity tone, anterior lower-extremity muscle wasting, lack of response to noxious stimuli in the lower extremities, diminished upper-extremity reflexes (1 +), and absence of lower-extremity reflexes and vibration/proprioception sensation. Two days later, her exam progressed to marginal finger movement and complete absence (0 +) of deep tendon reflexes in both upper and lower extremities.

Neurological deterioration was progressive throughout hospitalization. Initially, the patient retained sufficient upper-extremity function to remove her Dobhoff tube; over time, this declined to minimal finger movement, accompanied by worsening generalized weakness and diffuse areflexia. Given the patient’s progressive ascending weakness, areflexia, sensory deficits, and albuminocytologic dissociation on CSF analysis, an immune-mediated polyradiculoneuropathy was suspected. However, definitive confirmation was limited by the absence of electrophysiological studies.

An empiric 5-day course of intravenous immunoglobulin (IVIG) was initiated, consistent with established treatment recommendations for suspected Guillain–Barré syndrome [7]. On reevaluation two days later, the patient remained disoriented but reported cessation of hallucinations, began conversing in full English, demonstrated improved upper-extremity movement, and was able to wiggle her feet. Muscle tone improved, though she remained areflexic. The following day, she was oriented to person, place, and event, with strength of 2/5 in both upper and lower extremities, partial return of reflexes (1 +), and improving sensation.

Electrophysiological testing with NCS/EMG was not completed during hospitalization. The available record does not identify a single definitive reason for this omission; contributing factors may have included the patient’s altered mental status, limited cooperation with diagnostic testing, clinical prioritization of empiric immunotherapy, and early post-treatment improvement. The absence of NCS/EMG remains a major limitation because it prevents objective confirmation of GBS, classification of neuropathy subtype, and exclusion of axonal, vasculitic, or critical illness–related neuropathies.

Respiratory assessment data were also unavailable in the reviewed medical record. Specifically, measurements commonly used to monitor respiratory involvement in Guillain–Barré syndrome, including negative inspiratory force (NIF), forced vital capacity (FVC), and serial bedside respiratory function assessments, were not documented. Likewise, no evidence regarding the need for ventilatory support or intensive respiratory monitoring was available for review. The absence of these data limits assessment of disease severity and the potential extent of respiratory muscle involvement.

Following completion of IVIG therapy, the patient demonstrated substantial improvement in mental status, movement, and strength. According to family, she returned to her baseline mental status; however, residual extremity weakness persisted. She was discharged to a skilled nursing facility, where she remained for approximately one month. Due to insurance and transportation barriers, follow-up care was limited. Several months later, the patient unfortunately succumbed to sepsis secondary to a decubitus ulcer.

## Discussion

The patient’s clinical features—including progressive ascending weakness, areflexia, and albuminocytologic dissociation—are consistent with a suspected immune-mediated polyradiculoneuropathy such as Guillain–Barré syndrome; however, this interpretation must be considered in the context of incomplete diagnostic evaluation. Although no clearly documented antecedent respiratory or gastrointestinal infection was identified prior to admission, her presentation included subjective fevers, gastrointestinal symptoms (nausea, hematemesis, and endoscopy-confirmed gastritis), and a urinary tract infection during hospitalization. These findings raise the possibility of an underlying infectious or inflammatory process preceding the onset of neurological symptoms. While none of these factors can be definitively established as classical triggers, they may represent potential antecedent events contributing to immune activation. Notably, antecedent gastrointestinal infection is among the most commonly reported triggers of GBS. Although no specific pathogen was identified in this case, the patient's gastrointestinal illness and systemic symptoms raise the possibility of a preceding inflammatory or infectious process.

Applying the Brighton Collaboration diagnostic framework retrospectively, this case most closely meets Level 2 diagnostic certainty for Guillain–Barré syndrome. The patient demonstrated bilateral progressive weakness, decreased or absent deep tendon reflexes in the affected limbs, an apparently monophasic course with neurological deterioration followed by improvement, and cerebrospinal fluid albuminocytologic dissociation, with an elevated protein concentration of 126 mg/dL and a white blood cell count of 1/mm3. Electrophysiological studies were not performed, precluding Level 1 diagnostic certainty and preventing characterization of the underlying neuropathic process. Although electrophysiological confirmation was unavailable, current practice recommendations support prompt initiation of immunotherapy when Guillain–Barré syndrome is strongly suspected clinically, even while confirmatory investigations are pending [7]. Nevertheless, because the diagnostic evaluation remained incomplete, several competing diagnoses could not be definitively excluded, although no alternative explanation for the progressive peripheral neurological deficits was definitively established. Importantly, without the subsequent emergence of objective neurological deficits, the diagnostic evaluation may reasonably have remained focused on psychiatric or systemic causes of altered mental status. Accordingly, this case is best interpreted as a suspected immune-mediated polyradiculoneuropathy meeting Brighton Level 2 diagnostic certainty for Guillain–Barré syndrome, rather than as definitive confirmation of an atypical presentation of Guillain–Barré syndrome.

### Differential diagnosis and competing explanations

The patient’s presentation required consideration of several competing diagnoses capable of producing both neuropsychiatric manifestations and peripheral neurological deficits. Given the incomplete diagnostic evaluation, none of the following conditions could be definitively excluded, and each contributed to the diagnostic uncertainty surrounding this case.

#### Autoimmune encephalitis

Autoimmune encephalitis remains one of the strongest competing diagnoses because the patient demonstrated prominent psychiatric symptoms, hallucinations, altered consciousness, behavioral changes, and subsequent improvement following intravenous immunoglobulin therapy. However, brain MRI, electroencephalography (EEG), and serum and cerebrospinal fluid neuronal antibody testing—including antibodies such as anti-NMDAR, LGI1, CASPR2, GABABR, and AMPAR—were not performed. Consequently, autoimmune encephalitis cannot be meaningfully excluded. Furthermore, IVIG is an established treatment for autoimmune encephalitis; therefore, clinical improvement following immunotherapy cannot be interpreted as evidence favoring Guillain–Barré syndrome over autoimmune encephalitis. [16, 17 ]

Although both disorders may respond to IVIG, Guillain–Barré syndrome primarily affects the peripheral nervous system and classically presents with ascending weakness, areflexia, and albuminocytologic dissociation, whereas autoimmune encephalitis primarily involves the central nervous system and commonly presents with psychiatric symptoms, seizures, cognitive dysfunction, abnormal MRI or EEG findings, and neuronal autoantibodies [16]. In this patient, features of both syndromes were present, but the absence of MRI, EEG, and antibody testing precluded meaningful differentiation between these disorders.

#### Critical illness polyneuropathy

Critical illness polyneuropathy was also considered because generalized weakness and reduced reflexes may develop during severe systemic illness [18]. However, the patient’s neurological deterioration appeared to progress alongside evolving neuropsychiatric symptoms rather than after prolonged critical illness or extended intensive care unit admission. Nevertheless, in the absence of electrophysiological studies, critical illness polyneuropathy cannot be definitively excluded.

#### Rheumatoid arthritis-associated vasculitic neuropathy

The patient’s history of rheumatoid arthritis introduces additional diagnostic complexity and represents a major confounding factor in the diagnostic evaluation. Rheumatoid arthritis is associated with several forms of peripheral neuropathy, including immune-mediated vasculitic neuropathy and entrapment neuropathies, while immunosuppressive therapies commonly used to treat rheumatoid arthritis may also increase susceptibility to infection and other neurological complications [19]. Unfortunately, information regarding disease activity, inflammatory markers, rheumatologic serologies, immunosuppressive medication history, and evidence of systemic vasculitis was unavailable in the reviewed medical record, limiting assessment of these possibilities. Although the patient’s relatively symmetric ascending weakness, diffuse areflexia, sensory deficits, and albuminocytologic dissociation were more suggestive of an acute immune-mediated polyradiculoneuropathy than the asymmetric mononeuritis multiplex classically associated with rheumatoid vasculitic neuropathy, definitive distinction was not possible [19]. In the absence of electrophysiological studies, comprehensive rheumatologic evaluation, and, where clinically indicated, nerve biopsy, rheumatoid arthritis-associated neuropathy cannot be excluded and remains an important competing diagnosis.

#### Acute porphyria

Acute porphyria can present with neuropsychiatric symptoms, autonomic dysfunction, abdominal complaints, and motor neuropathy [20]. Although this diagnosis was not specifically investigated during hospitalization, the combination of psychiatric manifestations and progressive weakness warrants its inclusion within the differential diagnosis. The absence of porphyrin studies prevents exclusion of this disorder.

#### Metabolic and infectious encephalopathy

Altered mental status may also have resulted from metabolic or infectious encephalopathy. Potential contributing factors included urinary tract infection, gastrointestinal illness, hospitalization, and possible systemic inflammatory responses. Although routine laboratory testing was reportedly unremarkable relative to the patient’s clinical presentation, complete laboratory datasets—including inflammatory markers and detailed metabolic studies—were unavailable for independent review. Therefore, metabolic and infectious encephalopathy cannot be completely excluded.

#### Paraneoplastic neurological syndrome

Paraneoplastic neurological syndromes should also be considered given the patient’s age, subacute neuropsychiatric decline, and progressive peripheral neuropathy [21]. No malignancy screening, paraneoplastic antibody testing, or oncologic evaluation was available within the reviewed medical record. Consequently, a paraneoplastic immune-mediated neurological syndrome cannot be excluded.

#### Spinal cord pathology

Structural spinal cord pathology may produce progressive weakness and sensory abnormalities [22]. However, the presence of diffuse areflexia together with albuminocytologic dissociation favored a peripheral nervous system process over an isolated spinal cord lesion. Nevertheless, because comprehensive neuroimaging was not completed, spinal pathology could not be definitively excluded.

#### Medication-related encephalopathy or neuropathy

Medication-related neurological dysfunction was also considered. During hospitalization the patient received antipsychotic medication and lorazepam; however, her psychiatric symptoms preceded these treatments, making medication-induced encephalopathy an incomplete explanation for the overall clinical presentation. Nevertheless, medication effects may have contributed to fluctuations in mental status during hospitalization.

#### Heavy metal or toxic neuropathy

Heavy metal toxicity and other toxic neuropathies represent uncommon but recognized causes of combined psychiatric and peripheral neurological symptoms [23]. No toxicological investigations or heavy metal screening were performed during hospitalization. Although the clinical presentation was less suggestive of toxic neuropathy than immune-mediated disease, these etiologies cannot be completely excluded given the incomplete diagnostic evaluation.

### Clinical lessons

A central clinical lesson from this case is the critical importance of repeated, objective neurological examination in patients presenting with unexplained neuropsychiatric symptoms. At initial presentation, the patient’s psychiatric manifestations—including hallucinations, altered behavior, disorientation, and cognitive decline—could reasonably have been attributed to several competing diagnoses, including delirium, primary psychiatric illness, autoimmune encephalitis, metabolic encephalopathy, medication-related effects, or other systemic disorders. However, the subsequent emergence of objective neurological findings, including symmetrical ascending weakness, diffuse areflexia, sensory deficits, and progressive motor decline, fundamentally shifted the diagnostic framework toward peripheral nervous system involvement and justified consideration of Guillain–Barré syndrome or another immune-mediated neuropathy. Although the available evidence ultimately supports only a suspected immune-mediated polyradiculoneuropathy rather than a definitive diagnosis of GBS, this case demonstrates that careful serial neurological examinations may identify clinically significant peripheral nervous system involvement that is not apparent during initial evaluation. For patients with persistent or unexplained neuropsychiatric presentations, repeated neurological assessment remains essential to broadening the differential diagnosis, guiding appropriate consultation, and expediting potentially life-saving immunomodulatory treatment, including IVIG or plasma exchange when clinically indicated [7]. Without the emergence of objective neurological deficits, the diagnostic evaluation may reasonably have remained focused on psychiatric or systemic causes of altered mental status.

### Clinical implications and future directions

Despite the significant complication of altered mental status, the patient’s presentation would be atypical for delirium alone. Although uncommon, neuropsychiatric manifestations in GBS—including hallucinations and psychosis—have been described [9–11]. However, the early onset and prominence of psychiatric symptoms in this case, preceding and evolving alongside motor deficits, suggest a more complex pathophysiological interplay.

Reports describing this sequence of prominent neuropsychiatric symptoms preceding motor deficits, followed by rapid clinical improvement with IVIG and limited responsiveness to psychiatric medications, remain scarce [12]. This case therefore highlights the diagnostic challenges posed by atypical presentations of immune-mediated neurologic disease.

Early identification of such presentations is essential to reduce diagnostic delay and mitigate complications. Multidisciplinary management, including psychiatric care, rehabilitation, and social support, is critical for optimizing outcomes.

The intensity of depression in GBS has been shown to correlate with both anxiety levels and severity of motor weakness [8, 10,15 ]. Persistent psychological symptoms may impair rehabilitation and reduce quality of life. Therefore, long-term care for patients with GBS and concurrent psychiatric manifestations should incorporate a multidisciplinary approach, including psychotherapy, pharmacologic management, and physical rehabilitation. [14]

The patient’s eventual death from sepsis secondary to a decubitus ulcer represents an important adverse outcome and educational component of this case. Although causality cannot be established from the available record, persistent weakness, prolonged immobility, impaired access to follow-up care, and discharge to a skilled nursing facility likely increased vulnerability to pressure injury and infection. Evidently, access barriers, including transportation and insurance limitations, are recognized contributors to adverse health outcomes [13]. This outcome underscores that suspected GBS or immune-mediated neuropathy should not be viewed only as an acute diagnostic challenge, but also as a condition requiring aggressive prevention of immobility-related complications, coordinated rehabilitation, skin care, pressure-ulcer prevention, and reliable post-discharge neurological follow-up.

Overall, this case highlights the diagnostic tension between peripheral and central nervous system autoimmune processes in the setting of incomplete diagnostic data. While the patient’s clinical findings support an acute peripheral immune-mediated polyradiculoneuropathy such as GBS, the prominence of neuropsychiatric symptoms raises concern for a concurrent or alternative central process. Concurrent peripheral and central autoimmune processes were considered during diagnostic evaluation; however, available evidence was insufficient to establish such an overlap syndrome.

## Limitations

This case is subject to several important limitations. First, definitive diagnostic confirmation was limited by incomplete diagnostic testing. Electrophysiological studies (EMG/NCS) were not performed, preventing objective confirmation and characterization of the peripheral neuropathy. Brain MRI and electroencephalography (EEG) were unavailable because of persistent patient non-cooperation, precluding comprehensive evaluation for central nervous system pathology. In addition, the reviewed medical record did not document whether spinal MRI was performed, limiting assessment for structural spinal causes of the patient's neurological deficits. Autoimmune encephalitis-specific antibody testing was likewise unavailable, limiting exclusion of competing autoimmune disorders.

Second, several laboratory and clinical data were unavailable for independent review. Although routine laboratory studies were reportedly unremarkable relative to the patient's presentation, specific laboratory values—including inflammatory markers, hemoglobin, admission vital signs, and hemodynamic parameters—were unavailable, limiting assessment of potential metabolic, infectious, and inflammatory contributors.

Third, the patient's underlying rheumatoid arthritis could not be comprehensively evaluated because detailed information regarding disease activity, immunosuppressive medication history, rheumatologic serologies, malignancy screening, and paraneoplastic antibody testing was unavailable in the reviewed medical record. These omissions limited assessment of alternative autoimmune and paraneoplastic neurological disorders.

Finally, respiratory assessment data—including negative inspiratory force, forced vital capacity, serial respiratory monitoring, and documentation regarding the need for ventilatory support—were unavailable, limiting objective assessment of respiratory involvement and overall disease severity.

Collectively, these limitations preclude definitive distinction between Guillain–Barré syndrome, autoimmune encephalitis, rheumatoid arthritis-associated vasculitic neuropathy, critical illness neuropathy, paraneoplastic neurological syndromes, metabolic or infectious encephalopathy, spinal cord pathology, toxic neuropathies, or a potential overlap syndrome. Accordingly, this report should not be interpreted as establishing a definitive diagnosis of atypical Guillain–Barré syndrome, but rather as an educational case illustrating the diagnostic complexity of overlapping neuropsychiatric and peripheral neurological manifestations when definitive diagnostic testing is incomplete.

## Conclusion

This case illustrates the diagnostic challenge of evaluating prominent neuropsychiatric symptoms that evolve alongside objective peripheral nervous system findings. The emergence of symmetrical weakness, sensory loss, and areflexia was critical in prompting consideration of GBS or another immune-mediated polyradiculoneuropathy; however, autoimmune encephalitis, RA-associated neuropathy, paraneoplastic disease, metabolic encephalopathy, and other mimics could not be definitively excluded. The major lesson is not that neuropsychiatric symptoms were caused by GBS, but that repeated neurological examination and comprehensive ancillary testing are essential when psychiatric symptoms occur with evolving neuromuscular deficits. Treatment response to IVIG should be interpreted cautiously, and similar cases require early multidisciplinary evaluation, confirmatory electrophysiology, Central Nervous System (CNS) imaging, EEG, autoimmune/paraneoplastic testing, and careful prevention of immobility-related complications. This report should be interpreted as an educational case illustrating the diagnostic complexity of overlapping neuropsychiatric and peripheral neurological manifestations rather than as definitive evidence confirming an atypical presentation of Guillain–Barré syndrome.

## Supplementary Information

Supplementary Material 1.

## Data Availability

No datasets were generated or analyzed during the current study.
